# Operational, molecular, and cultural perspectives at the Neurological Foundation Human Brain Bank, New Zealand

**DOI:** 10.3389/fneur.2026.1900088

**Published:** 2026-07-27

**Authors:** Blake Highet, Malvindar Singh-Bains, Marika Eszes, Remai Parker, Emma McShane, Catherine Webb-Robinson, Kristina Hubbard, Frances Biggins, Shruti Sharma, Paige Bell, Oliver Burnett, Klaus Lehnert, Jessie C. Jacobsen, Brigid Ryan, Mindy Hu, Clinton Turner, Richard L. M. Faull, Maurice A. Curtis

**Affiliations:** 1 Centre for Brain Research, Faculty of Medical and Health Sciences, Department of Anatomy and Medical Imaging, University of Auckland, Auckland, New Zealand; 2 Faculty of Science, School of Biological Sciences, University of Auckland, Auckland, New Zealand; 3 LabPlus, Auckland City Hospital, Auckland, New Zealand

**Keywords:** Alzheimer’s disease, brain banking, brain donation, community engagement, indigenous health, neuropathology, New Zealand, Parkinson’s disease

## Abstract

Post-mortem human brain tissue is an irreplaceable resource for advancing understanding of the brain and neurological disease. Brain banks that collect, preserve, and distribute this tissue underpin discoveries across neuropathology, genomics, and emerging molecular disciplines. Yet brain banking is a resource-intensive enterprise that faces distinct operational, cultural, and scientific challenges depending on the national context in which it operates. Here, we present the perspective of the Neurological Foundation Human Brain Bank (NFHuBB) — New Zealand’s sole post-mortem human brain bank, based at The University of Auckland. Operating as the only such facility in the country, the NFHuBB confronts a unique convergence of challenges: the logistical complexity of coordinating donation across a dispersed population, the scientific imperative to preserve tissue for molecular-era multi-omics technologies, and a multi-cultural population that needs culturally informed consent practices. We describe the growth of the bank’s donor registry and collection over 45 years with specific emphasis on donations in the last five calendar years (2021–2025), outline recent advances in tissue processing, including the development of formalin-fixed paraffin-embedded (FFPE) RNA preservation protocols and integration of whole-genome sequencing from un-fixed cerebellar tissue. Finally, we discuss the community engagement strategies that have supported increasing donation rates in New Zealand.

## Introduction

The post-mortem human brain is the gold standard resource for studying and understanding the cellular and molecular basis of neurological disease. Because no animal model fully recapitulates the complexity of human brain diseases, such as Alzheimer’s disease (AD), Parkinson’s disease (PD), Huntington’s disease (HD), or the spectrum of rarer conditions, brain banks play a critical role in collecting, characterizing, storing, and distributing brain tissue for research. Furthermore, brain banks strengthen ethical and legal frameworks for donation by setting clear consent, privacy and governance standards for donation.

Globally, brain banking has faced persistent challenges. Donation rates remain low relative to the need in most countries; the molecular integrity of archived tissue is often inadequate for contemporary genomic and transcriptomic assays; and most countries prohibit advertising organ donation, which hampers sustainable donor recruitment at scale (1). These challenges are well documented by large, well-resourced brain bank networks in the United Kingdom (2), the United States (3), and Europe (4). Less well-described are the challenges facing brain banks operating as sole national providers in small countries, where the consequences of operational failure are magnified, community relationships are more proximate, and the research infrastructure and government funding for research is modest.

The Neurological Foundation Human Brain Bank (NFHuBB), henceforward called ‘the brain bank’, based in the Department of Anatomy and Medical Imaging, and Centre for Brain Research at The University of Auckland, is New Zealand’s only post-mortem brain bank. Formally established in 1994 with support from the Neurological Foundation of New Zealand, the brain bank has grown to become a nationally significant research resource, having received tissue from >1,000 donors spanning a range of neurological conditions. In this perspective, we reflect on more than 30 years of operation as a formalized brain bank and describe four interconnected challenges that define our brain bank’s experience: (1) the operational and scientific challenge of maintaining and growing a national collection; (2) the molecular challenge of preserving and characterizing tissue for future neuropathology investigation; (3) the community challenge of building a culture of brain donation in a multicultural nation with diverse and evolving attitudes toward post-mortem tissue research and finally; (4) working with other countries where brain banking initiatives are in their germinal phase to advance the global neuroscience capacity. We offer these reflections as a practitioner perspective intended to contribute to the broader conversation about the future of brain banking globally.

### New Zealand’s sole brain bank: growth and operational realities

**Figure 1:**
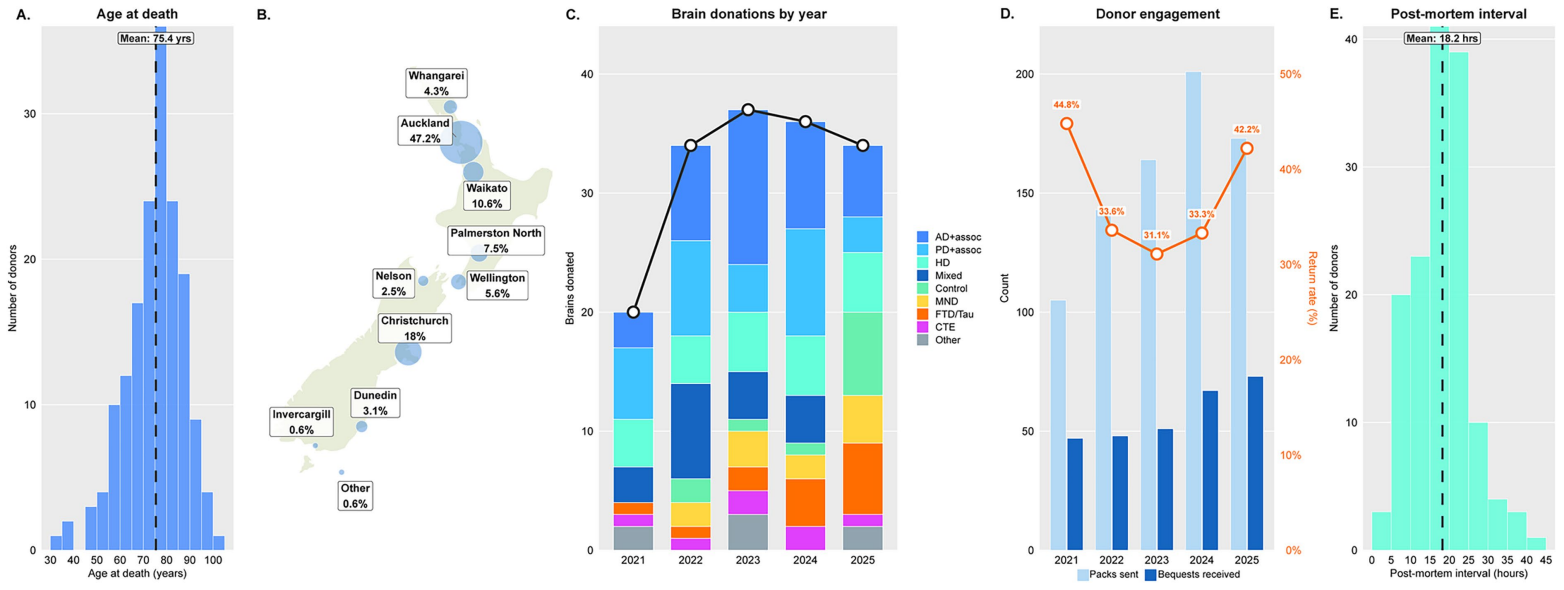
Brain donations to the Neurological Foundation Human Brain Bank in the last five years. **(A)** The average age at donation of our brain donors over the last five years is 75.4 years (range 33–102 years). **(B)** Regional breakdown of brain donations received across New Zealand in the last five years. **(C)** Disease breakdown of brain donations over the last five years. Note that not all pathologies have been confirmed for cases donated in 2025. **(D)** Donor engagement over the last five years. Light blue indicates donor packs sent out, dark blue indicates completed returns, and the orange line shows the percentage of donor packs completed and returned each year. **(E)** The average post-mortem interval of our brain donors over the last five years is 18.2 h (range 2.5–43 h). AD, Alzheimer’s disease; PD, Parkinson’s disease; HD, Huntington’s disease; MND, motor neuron disease; FTD, fronto-temporal dementia, CTE, chronic traumatic encephalopathy.

The brain bank received its first brain in January 1981 when Huntington’s disease (HD)- affected families asked researchers at the University to examine their loved ones’ brains after death. Prior to the discovery of the HD gene in 1993, diagnosis relied on clinical symptoms that could be confused with other movement disorders, making neuropathological confirmation invaluable to families. These families then generously donated brain tissue, asking researchers to continue to use it for research into disease causation and treatment. The success of early HD studies led to the expansion of the programme to include PD, AD, and amyotrophic lateral sclerosis (ALS) / motor neuron disease (MND), rarer brain diseases, and neurologically normal cases. Over the last five years, the average age at death of our donors is 75 years old (range 33–102), with 61% of our donors being male and the remaining 39% being female (Figure 1A). Donations have been received from across New Zealand, with the majority of donors located in the Auckland region (47.2%), followed by donors from Christchurch (18%) and the Waikato (10.6%, Figure 1B). Annual donation numbers with disease representation by year is shown in Figure 1C. Donor engagement has trended positively (Figure 1D). Donor information packs distributed nationally yielded a return rate of approximately 37% over the last 5 years: 786 donor packs were sent to those who requested them and we received 286 completed donor registration forms back. This increasing donor engagement reflects growing community awareness of and willingness to support brain donation, as well as strengthened referral networks with neurologists and geriatricians across New Zealand. Specific longitudinal studies have also led to greater interest in brain donation by participants enrolled in studies such as the NZP3 study on Parkinson’s progression (5), the NZ genetic frontotemporal dementia study FTDGeNZ (6), and the Dementia Prevention Research Clinic participants (7).

New Zealand presents a distinctive operational context for brain banking. With a population of approximately five million dispersed across two main islands, and a healthcare system organized around regional hospitals (now Health New Zealand | Te Whatu Ora), coordinating post-mortem brain donation is a geographically complex undertaking reliant on local mortuaries to remove the brain and courier it to Auckland for processing and storage. The NFHuBB is the singular repository for donated neurological tissue in the country, carrying an attendant responsibility to receive tissue from all of New Zealand and to serve researchers from all New Zealand institutions equitably. Furthermore, the brain bank relies on charitable funding from the Neurological Foundation for support.

Post-mortem interval (PMI) management, a critical determinant of tissue quality, requires informed consent from next of kin, a promptly signed death certificate and other documents, as well as a rapid response from mortuary services that may be located at a significant distance from Auckland. The brain bank system runs separately to the national organ donation process that exists to receive organs for transplantation. Therefore, the brain bank has developed relationships with hospital mortuary services across both the North and South Islands to facilitate PMI times of less than 24 h, though geographic constraints mean PMI variability remains a challenge for cases arising outside major urban centers. Over the last five years, our average PMI is 18 h (range 2.5–43 h, Figure 1D). To facilitate the transport of the body of a brain donor to the nearest mortuary, we rely on the wonderful and vital cooperation from the funeral director of the family’s choosing.

Furthermore, as the sole custodian of New Zealand’s post-mortem neurological tissue resource, the brain bank bears a unique curatorial responsibility. This has driven investment in robust storage infrastructure and in the development of novel tissue preservation and characterization approaches described in the following section.

A key component of the brain bank’s operational model is the systematic retrospective collection of detailed clinical histories for all donors. Staff are employed (usually medical students) to liaise directly with general practitioners and family physicians across New Zealand to retrieve comprehensive clinical records for each donor. This initiative, embedded within the bank’s standard operating procedures, ensures that post-mortem tissue can be meaningfully contextualized with ante-mortem clinical trajectory, including diagnosis date, medication history, comorbidities, and functional decline. Where a brain donor has been enrolled in one of New Zealand’s longitudinal studies of neurological disease, e.g., FTDGeNZ (6) or NZP3 (5), we request access to the study data on that individual from the next of kin and from the study leads so that clinical assessments and imaging can be compared to post-mortem samples. The resulting clinical data substantially increases the scientific value of donated tissue, enabling researchers to stratify cases by disease stage, symptom profile, treatment exposure, or comorbidity profile. This model of student-supported clinical record retrieval represents a pragmatic and scalable solution to a challenge faced by many smaller brain banks, ensuring that donated tissue is accompanied by the depth of clinical annotation necessary for high-quality translational research.

## Advancing tissue processing for the molecular era

Brain banks have historically operated on a standard workflow: fresh dissection at autopsy, bisection of the brain, snap-freezing of one hemisphere for biochemical and molecular studies, and formalin fixation of the other for neuropathological assessment and archival storage (or fixation of the whole brain). However, the emergence of spatially-resolved transcriptomics, single-cell sequencing, single-nucleus RNA sequencing, and other nucleic acid-dependent platforms has fundamentally altered the demands placed on archived tissue and has exposed a critical weakness in existing archival collections worldwide where tissue has not been optimally stored for molecular analysis.

Formalin-fixed paraffin-embedded (FFPE) tissue, the format in which the majority of the world’s archived neuropathological material exists, undergoes extensive RNA degradation and crosslinking during fixation and long-term storage. For brain banks like ours with decades of archival material, this represents both a challenge and an opportunity: if RNA integrity can be adequately preserved, the research value of existing collections is dramatically expanded. In response to this challenge, we have developed a modified FFPE tissue storage approach, leaving tissue blocks awaiting paraffin embedding in Universal Molecular Fixative (UMFIX) rather than formalin, to preserve RNA integrity in archival post-mortem brain samples as highlighted by a ~ 32% point increase in DV_200_ score: the total percentage of RNA at least 200 nucleotides in length (cite Highet et al., under revisions). These fixation methods have enabled molecular-grade preservation for multi-omic interrogation, including spatially resolved transcriptomic profiling using platforms such as 10x Genomics Visium HD. We encourage other banks to adopt fixation and storage methods that readies prospective collections for molecular studies such as spatial transcriptomics.

One of the most significant recent advances at the brain bank has been the integration of whole-genome sequencing (WGS) into the standard tissue-processing workflow. Historically, the brain banking field has been slow to adopt systematic genomic characterization of donors, with the result that large archival collections exist without accompanying genetic data. This is a significant limitation as genome-wide association studies (GWAS) and polygenic risk score analyses, and selection of donor tissue to include or exclude ‘background’ genetic mutations relevant to the condition, become central tools in neurological disease research. At our brain bank, we have addressed this gap through a protocol modification that leverages the existing mortuary network that underpins our tissue collection.

At the time of brain removal, mortuary staff are asked to dissect and separately preserve a sample of fresh cerebellar tissue regardless of where in New Zealand the brain is coming from so we can genotype every case. The cerebellum is selected for this purpose for several practical reasons: 1, it is relatively uniform in cellular composition throughout the folia, 2, it is not the primary tissue of interest in most neuropathological studies of the human brain, 3, it is an abundant tissue and rarely all of it gets used in cerebellar research studies, 4, it yields high-quality, high-molecular-weight DNA suitable for WGS. Our samples are sequenced to a mean depth of 34.1x (range 29–41.7x), exceeding the ~30x threshold recommended for confident germline variant detection. This means that the cerebellar sample can be dedicated to genomic analysis without compromising the cerebral hemispheric tissue available for standard neuropathological and molecular research programmes.

The resulting WGS data provide a permanent genetic record for each donated case, enabling researchers to: identify known, causative, pathogenic variants in genes such as *LRRK2*, *GBA*, *SNCA* (PD) (8), *APP*, *PSEN1*, *PSEN2*, *APOE* (AD) (9), and *HTT* (HD) (10); calculate polygenic risk scores for sporadic disease, and stratify cases by genetic background for transcriptomic and proteomic analyses. The integration of genomic data with post-mortem neuropathological characterisation positions the brain bank’s collection as a genuinely multi-modal resource, aligning it with the data standards increasingly expected by international collaborators and funding bodies. WGS data, once generated, provide a permanent resource that grows in value as analytical methods advance and reference datasets expand. Similar cerebellar sampling protocols could be readily adopted by other brain banks seeking to add genomic depth to their collections without major disruption to existing workflows.

Genetic results are provided to researchers upon reasonable request alongside tissue access, enabling stratification by known polymorphisms to enhance scientific output. Strict conditions of supply govern data use: results must not be shared or used in any way that could identify individuals or their relatives. Data is stored on secure hard drives and network infrastructure. The brain bank operates on the general principle that genetic information is not routinely returned to donor families. However, if a potentially clinically actionable polymorphism is identified, neurologists and geneticists associated with the brain bank would disclose the finding to the family. In such events, the family receives a disclosure of the findings and is referred to a clinical genetics service for confirmatory testing and genetic counseling. To date, this disclosure protocol has not been triggered.

This management process for incidental findings was reviewed and approved by the Health and Disability Ethics Committee (HDEC). Regarding informed consent, the brain bank does not utilize an opt-in/out framework for specific genetic substudies. Consent for genetic testing is obtained from all donor families at the time of brain donation. Bioinformatics and genetic variant identification are performed by a dedicated geneticist, with the Research and Education Advanced Network New Zealand (REANNZ) providing computing resources and secure data storage. This approach also facilitates secure sharing of data with approved collaborators.

From our WGS data we do not share raw data or large haplotypes of called variants. We do not allow lineage analysis and as much as possible use the WGS data to better inform and support neuropathological studies being performed by researchers using human tissue samples. Brain banks acquiring WGS information need to have processes and procedures in place for when an incidental tractable findings from WGS emerge.

## Increasing brain donation in New Zealand

Organ donation rates in New Zealand remain low, which means rarer diseases are often not represented in the brain bank donations, although our donation rates as a proportion of the population are comparable to other well-established, international brain banks. Even for more common neurodegenerative diseases, after stratification of cases for age, comorbidities, sex, and PMI, there are often insufficient cases to constitute a substantial study or at least compromises need to be made around case selection—matching neurologically normal tissues to the disease groups can be even harder. To increase brain donor rates, we have taken the following approaches:

1. A partnership approach between researchers, potential donors, and their families. The brain bank team engage directly with interested individuals by phone and email, and coordinate with residential care facilities to enable timely brain collection after death. We also contact next of kin after each donation to provide pathology results, so families understand the precise details of their loved one’s diagnosis.
2. For many Māori (the indigenous people of New Zealand), the head (upoko) is considered sacred (tapu), and practices surrounding death are governed by customs and protocols (tikanga Māori) that vary across tribes (iwi) and regions. This does not preclude brain donation; rather, it demands engagement that is culturally informed, respectful of tikanga, and oriented toward reciprocal benefit. In addition, to many indigenous people, genomic information is more sensitive than other types of information and should be managed with care and it’s usage should be determined in consultation with members of that ethnic group. For our brain bank, donor information is available in te reo Māori on our website, and Māori community officers can communicate with potential donors by phone or in person.
3. The brain bank works closely with community organisations including Alzheimer’s Auckland, Alzheimer’s New Zealand, Epilepsy NZ, Huntington’s Disease Associations of New Zealand, Motor Neurone Disease Association NZ, and Parkinson’s New Zealand. Broader awareness initiatives include the annual Brain Bee secondary school competition, public lectures, media engagement, and joint campaigns with the Neurological Foundation of New Zealand. Collectively, these efforts build community familiarity with brain research and donation, and identify potential donors who may not access the bank through clinical pathways.

### NFHuBB in an international network of brain banks

The brain bank has strong linkages to international brain research infrastructure through global networks. One example is the Concussion Legacy Foundation (CLF) Global Brain Bank, integrating New Zealand-based tissue resources into collaborative studies on neurodegeneration and repetitive-head-injury disease like chronic traumatic encephalopathy (CTE).

Another example is that our well-established brain banking protocols have also been adopted by international partners of large multi-institutional collaborations to ensure the greatest tissue quality. A recent collaboration with the Collaborative Centre for X-Linked Dystonia-Parkinsonism (CCXDP) at Massachusetts General Hospital and the Sunshine Care Foundation in Panay, Philippines has resulted in establishment of the first human brain bank in the Philippines based on protocols optimized in our brain bank (11–13). Establishing this facility involved in-person visits to the Philippines to demonstrate perfusion and dissection protocols. Our involvement enabled high-quality tissue preparation, as shown by strong histological staining, good RNA integrity, clear neuronal transcript detection by RNA *in situ* hybridization, and successful western blotting. In-person engagement with Filipino researchers, clinicians, and community advocates helped build the trust needed to establish a culturally appropriate brain banking pipeline for XDP families. This experience may provide a model for developing brain banks for rare diseases in indigenous populations worldwide.

The NFHuBB provides brain tissue to international researchers within a tightly regulated framework of ethical oversight and donor consent. Families explicitly consent to domestic or international use of tissue; no commercial sale is permitted under New Zealand law. Access is granted on the basis of scientific merit and adherence to governance and data-sharing policies.

## Discussion: Lessons from the NFHuBB

The challenges described here — operational isolation, molecular preservation of tissue, and culturally informed community engagement — are not unique to New Zealand, but are experienced in concentrated form by small, sole-provider banks. The solutions developed at NFHuBB have, by necessity, been pragmatic, cost-effective, and designed for transferability.

From a funding and outreach perspective, having a strong relationship with the funder has been vital. In our case, the Neurological Foundation of New Zealand has been the major financial supporter of the brain bank since 1994. The relationship now operates more like a partnership, and the outreach to the public about brain donation is driven by the brain bank team and actively supported by the Neurological Foundation. While various funding models have been used over the years, the current model operates under a memorandum of agreement (an enduring document) and then a budget cycle of two years. The long-term support from the Neurological Foundation of New Zealand has been critical in giving funding security and enabled the brain bank to grow in terms of brain donation numbers and also in the types of techniques that tissue can be prepared for. Also, the establishment of a university brain bank advisory board chaired by the directors and comprising representatives of the donor families, community support neurological organisations, neurologists, research user groups and funders, provides oversight to ensure the brain bank responds quickly to changing needs of stakeholders.

Looking forward, we identify several priorities for the brain bank and for the global brain banking community more broadly. First, the integration of WGS as a routine component of tissue accession, using simple fresh tissue sampling protocols such as the cerebellar approach described here, should be considered standard practice for banks of sufficient scale. Even if the WGS is done at a later time, the marginal cost of cerebellar collection is low; the long-term scientific return is substantial. Second, the adoption of molecular-grade tissue-preservation protocols for FFPE material should become a standard expectation at the point of accession rather than an afterthought. Third, the development of culturally appropriate donation frameworks, particularly for indigenous communities, is an ethical and scientific priority that extends well beyond New Zealand.

Finally, we see opportunity in regional collaboration. New Zealand’s geographic position in the South Pacific places it within reach of communities, including Pacific Island nations with distinct genetic and epidemiological profiles, who are currently largely absent from global brain banking collections. A South Pacific brain banking network, anchored at the brain bank and developed in partnership with Pacific and Asian health researchers and communities, represents a long-term aspiration that would both expand the scientific reach of the bank and address a significant gap in the global representation of neurological disease tissue.

In conclusion, our experience demonstrates that world-class brain banking is achievable in a small, geographically isolated nation. It requires deliberate innovation in tissue processing, sustained community partnership, and advocacy for the long-term financial support that the curation of a national research resource demands. We offer these reflections as a contribution to the global conversation about how brain banks can best serve science and society in the decades ahead.

## Data Availability

The original contributions presented in the study are included in the article/supplementary material, further inquiries can be directed to the corresponding author.

## References

[1] Kretzschmar H. Brain banking: opportunities, challenges and meaning for the future. Nat Rev Neurosci. (2009) 10:70–8. doi: 10.1038/nrn2535, 19050713

[2] Keogh MJ Wei W Wilson I Coxhead J Ryan S Rollinson S . Genetic compendium of 1511 human brains available through the UK Medical Research Council brain banks network resource. Genome Res. (2017) 27:165–73. doi: 10.1101/gr.210609.116, 28003435 PMC5204341

[3] Hulette CM. Brain banking in the United States. J Neuropathol Exp Neurol. (2003) 62:715–22. doi: 10.1093/jnen/62.7.715, 12901698

[4] Bell JE Alafuzoff I Al-Sarraj S Arzberger T Bogdanovic N Budka H . Management of a twenty-first century brain bank: experience in the BrainNet Europe consortium. Acta Neuropathol. (2008) 115:497–507. doi: 10.1007/s00401-008-0360-8, 18365220

[5] MacAskill MR Pitcher TL Melzer TR Myall DJ Horne KL Shoorangiz R . The New Zealand Parkinson’s progression programme. J R Soc N Z. (2023) 53:466–88. doi: 10.1080/03036758.2022.2111448, 39439968 PMC11459764

[6] Ryan B O’Mara Baker A Ilse C O'Mara Baker A Brickell KL Kersten HM . The New Zealand genetic frontotemporal dementia study (FTDGeNZ): a longitudinal study of pre-symptomatic biomarkers. J R Soc N Z. (2023) 53:511–31. doi: 10.1080/03036758.2022.2101483, 39439966 PMC11459842

[7] Tippett LJ Cawston EE Morgan CA Melzer TR Brickell KL Ilse C . Dementia prevention research clinic: a longitudinal study investigating factors influencing the development of Alzheimer’s disease in Aotearoa, New Zealand. J R Soc N Z. (2023) 53:489–510. doi: 10.1080/03036758.2022.2098780, 39439970 PMC11459802

[8] Bloem BR Okun MS Klein C. Parkinson’s disease. Lancet. (2021) 397:2284–303. doi: 10.1016/S0140-6736(21)00218-X, 33848468

[9] Scheltens P Strooper BD Kivipelto M De Strooper B Holstege H Chételat G . Alzheimer’s disease. Lancet. (2021) 397:1577–90. doi: 10.1016/S0140-6736(20)32205-4, 33667416 PMC8354300

[10] Walker FO. Huntington’s disease. Lancet. (2007) 369:218–28. doi: 10.1016/S0140-6736(07)60111-1, 17240289

[11] Fernandez-Cerado C Legarda GP Velasco-Andrada MS Aguil A Ganza-Bautista NG Lagarde JBB . Promise and challenges of dystonia brain banking: establishing a human tissue repository for studies of X-linked dystonia-parkinsonism. J Neural Transm. (2021) 128:575–87. doi: 10.1007/s00702-020-02286-9, 33439365 PMC8099813

[12] Waldvogel HJ Bullock JY Synek BJ Curtis MA van Roon-Mom WMC Faull RLM. The collection and processing of human brain tissue for research. Cell Tissue Bank. (2008) 9:169–79. doi: 10.1007/s10561-008-9068-1, 18357514

[13] Waldvogel HJ Curtis MA Baer K Rees MI Faull RLM. Immunohistochemical staining of post-mortem adult human brain sections. Nat Protoc. (2006) 1:2719–32. doi: 10.1038/nprot.2006.354, 17406528

